# State of the Art in the Current Management and Future Directions of Targeted Therapy for Differentiated Thyroid Cancer

**DOI:** 10.3390/ijms23073470

**Published:** 2022-03-23

**Authors:** Horatiu Silaghi, Vera Lozovanu, Carmen Emanuela Georgescu, Cristina Pop, Bogdana Adriana Nasui, Adriana Florinela Cătoi, Cristina Alina Silaghi

**Affiliations:** 1 Department of Surgery V, “Iuliu Hatieganu” University of Medicine and Pharmacy Cluj-Napoca, 8 Victor Babes Street, 400012 Cluj-Napoca, Romania; hsilaghi@yahoo.com; 2 County Clinical Emergency Hospital Cluj, 3-5 Clinicilor Street, 400006 Cluj-Napoca, Romania; lozovanu.vera9@gmail.com; 3 Department of Endocrinology, “Iuliu Hatieganu” University of Medicine and Pharmacy Cluj-Napoca, 8 Victor Babes Street, 400012 Cluj-Napoca, Romania; c_e_georgescu@yahoo.com (C.E.G.); alinasilaghi@yahoo.com (C.A.S.); 4 Department of Pharmacology, Physiology, and Pathophysiology, Faculty of Pharmacy, “Iuliu Hatieganu” University of Medicine and Pharmacy Cluj-Napoca, 6A Louis Pasteur Street, 400349 Cluj-Napoca, Romania; 5 Department of Community Health, “Iuliu Hatieganu” University of Medicine and Pharmacy Cluj-Napoca, 6 Louis Pasteur Street, 400349 Cluj-Napoca, Romania; adriana.nasui@umfcluj.ro; 6 Department of Pathophysiology, “Iuliu Hatieganu” University of Medicine and Pharmacy Cluj-Napoca, 8 Victor Babes Street, 400012 Cluj-Napoca, Romania; adriana.catoi@umfcluj.ro

**Keywords:** differentiated thyroid cancer, radioactive iodine-refractory, targeted therapy, tyrosine kinase inhibitor, PI3K/AKT/mTOR pathway, MAPK pathway, immunotherapy, redifferentiation therapy, gene rearrangements

## Abstract

Two-thirds of differentiated thyroid cancer (DTC) patients with distant metastases would be classified as radioactive iodine-refractory (RAIR-DTC), evolving into a poor outcome. Recent advances underlying DTC molecular mechanisms have shifted the therapy focus from the standard approach to targeting specific genetic dysregulations. Lenvatinib and sorafenib are first-line, multitargeted tyrosine kinase inhibitors (TKIs) approved to treat advanced, progressive RAIR-DTC. However, other anti-angiogenic drugs, including single targeted TKIs, are currently being evaluated as alternative or salvage therapy after the failure of first-line TKIs. Combinatorial therapy of mitogen-activated protein kinase (MAPK) and phosphoinositide 3-kinase (PI3K) signalling cascade inhibitors has become a highly advocated strategy to improve the low efficiency of the single agent treatment. Recent studies pointed out targetable alternative pathways to overcome the resistance to MAPK and PI3K pathways’ inhibitors. Because radioiodine resistance originates in DTC loss of differentiation, redifferentiation therapies are currently being explored for efficacy. The present review will summarize the conventional management of DTC, the first-line and alternative TKIs in RAIR-DTC, and the approaches that seek to overcome the resistance to MAPK and PI3K pathways’ inhibitors. We also aim to emphasize the latest achievements in the research of redifferentiation therapy, immunotherapy, and agents targeting gene rearrangements in advanced DTC.

## 1. Introduction

Thyroid cancer (TC) is the most common endocrine neoplasia, accounting for 3.4% of all cancers diagnosed annually [1]. The incidence of TC has significantly increased in the last three decades. TC is predicted to be the second-leading cancer diagnosed in women and the ninth-leading in men by 2030 in the United States [2]. 

Papillary TC (PTC) and follicular TC (FTC) are the most common subtypes of TC, reaching up to 90–95% of all cases, merging into the distinct category of differentiated TC(DTC) [3]. DTCs are generally slow-growing tumors carrying an excellent prognosis with a 20-year overall survival (OS), greater than 90% after conventional treatment [4,5].

In most DTC cases, the standard management is effective and includes surgery, usually followed by radioactive iodine (RAI) remnant ablation, risk-stratified surveillance, and thyroid-stimulating hormone (TSH) suppression therapy [6]. However, local recurrence and distant metastases can occur in up to approximately 20% and 10% of cases, respectively, in the first ten years postoperatively [4]. The conventional therapeutic approaches for these patients include RAI therapy, surgical resection of the metastases, and external beam radiotherapy [4,6]. During the treatment, two-thirds of DTCs may become refractory to radioactive iodine (RAI) therapy, with a significant negative impact on prognosis and life expectancy [7]. The 10-year survival rate then drops to about 20% [8].

In the last few decades, the development of genome sequencing has made much progress in unravelling the molecular mechanisms underlying TC [9]. Most TCs harbor dysregulations involving the mitogen-activated protein kinase (MAPK) and phosphatidylinositol-3 kinase/mammalian target of rapamycin/protein-kinase B (PI3K/mTOR/Akt) signaling pathways. These pathways play a central part in the regulation of cellular proliferation, sending mitogen and proliferative signals from the cell membrane to the nucleus [10,11]. 

MAPK hyperactivation is pivotal in PTC initiation through point mutations of the BRAF oncogene. BRAF is a member of the RAF family of serine/threonine protein kinases downstream of RAS, mutated with a higher prevalence in PTC, ranging from 29 to 83% [12,13,14,15]. The mutation of BRAF activates the downstream transcription factors, leading to cell growth, differentiation, proliferation, and apoptosis. Different alterations of BRAF have been identified; however, most classic PTCs harbor the BRAFV600E variant [16]. Several studies claimed a correlation between the V600E variant and aggressive disease features, such as metastatic disease, invasion, and recurrence [17]. BRAFV600E oncogene induces TGF-beta secretion that inhibits sodium iodide symporter (NIS) expression, thus leading to resistance of TC to RAI therapy [18].

In turn, PI3K/mTOR/Akt pathway activation is crucial in FTC development. It can be triggered by activating mutations of RAS, PIK3CA, and AKT1 oncogenes and by inactivating the phosphatase and tensin homolog (PTEN), which negatively regulates this pathway. 

RAS is a family of GTP-binding proteins that acts through the MAPK and PI3K-AKT signaling pathways. Activating RAS mutations are found more frequently in FTC patients (28–68%), in up to 43% of follicular-variant PTCs (FVPTCs) [19], and in up to 47% of all non-invasive FVPTCs [20], showing the limited role for RAS mutations alone in the clinical outcomes of TC [21]. 

TC progression and dedifferentiation to poorly differentiated TC (PDTC) and anaplastic TC (ATC) involves several additional mutations affecting other signaling pathways, such as p53 and Wnt/β-catenin. More recently, telomerase reverse transcriptase (TERT) promoter mutations have been described in all histological subtypes of TC, with a significantly higher prevalence in aggressive and undifferentiated tumors, indicating their role in TC progression [22].

With increasing knowledge of the molecular pathogenesis of TC, the focus of cancer therapy has shifted from the treatments based on type and histology to those targeting specific gene dysregulations. Thus, novel targeted therapies are being developed for the subset of patients with a more aggressive disease course [23].

The present review will analyze and layout conventional management in DTC, first-line tyrosine kinase inhibitors (TKIs) in advanced, metastatic radioactive iodine refractory DTC (RAIR-DTC), alternative pathways to overcome the resistance to MAPK and PI3K pathways’ inhibitors, the most recent research breakthroughs in redifferentiation therapy for RAIR-DTC, as well as perspectives in immunotherapy and therapy targeting gene rearrangements for DTC.

## 2. Materials and Methods

PubMed, Embase, Scopus, and Google Scholar were searched for English-written articles to identify the latest guidelines, pre-clinical, and clinical studies on the conventional treatment of DTC, and new perspectives in the therapy of advanced, metastatic RAIR-DTC. The search was confined to manuscripts published from January 2000 to December 2021. The search strategy relied on a combination of key terms such as: “differentiated thyroid cancer”, “radioactive iodine-refractory differentiated thyroid cancer”, “targeted therapy”, “tyrosine kinase inhibitors”, “PI3K/AKT/mTOR pathway”, “MAPK pathway”, “immunotherapy”, “redifferentiation therapy”, and “gene rearrangements”.

## 3. Conventional Management of DTC

### 3.1. Surgical Treatment

Surgical resection of the primary tumor and clinically significant lymph node metastases remain the cornerstone of initial therapy in TC [4,24]. Effective initial surgery minimizes the risk of recurrence, improves disease-specific survival (DSS), facilitates postoperative RAI therapy, if indicated, and enables accurate staging and risk stratification [24].

**Figure 1:**
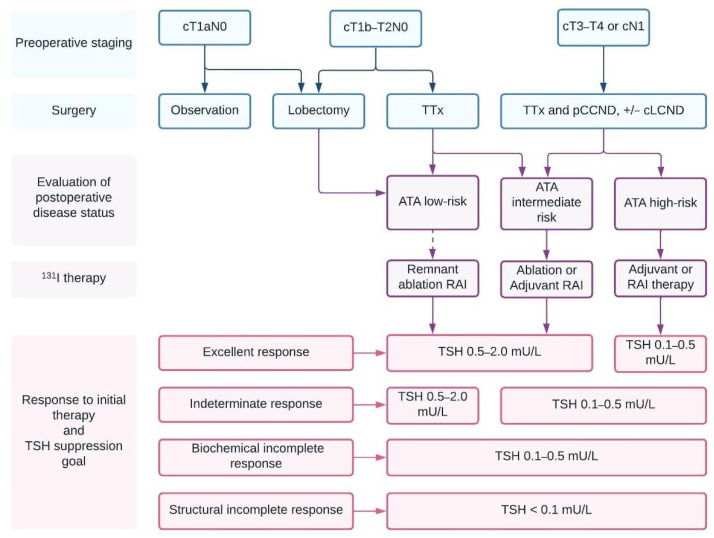
Initial management of radioiodine-avid differentiated thyroid cancer. The chart was created using the website www.lucidchart.com, accessed on 22 January 2022. Abbreviations: ATA = American Thyroid Association; cLCND = curative lateral compartment node dissection; pCCND = prophylactic central compartment node dissection; TSH = thyroid stimulatory hormone; RAI = radioiodine; TTx = total thyroidectomy.

Most guidelines recommend total thyroidectomy and gross removal of the primary tumor in patients with DTC greater than 4 cm or with gross extrathyroidal extension (ETE), cT4, clinically apparent nodal (cN1), or distant (cM1) metastatic disease (Figure 1). This approach is associated with higher disease-free survival (DFS), covers the potential multicentricity, allows the use of RAI as a diagnostic and therapeutic tool, and facilitates the monitoring of thyroglobulin (Tg) as a marker of recurrence and persistence of the disease [4,6,25].

The extent of the initial surgery in low-risk TC measuring 1 cm to 4 cm without ETE, and without clinical evidence of any lymph node metastases (cN0), is an area of debate between the bilateral procedure or the lobectomy, as recommended by the American Thyroid Association (ATA), and the National Comprehensive Cancer Network (NCCN) guidelines (Figure 1) [4,22]. Both approaches show similar survival outcomes [26,27,28]. However, total thyroidectomy has the advantage of being associated with a slightly lower risk of recurrence [28]. On the other hand, lobectomy has been associated twice less often with surgical complications (e.g., recurrent laryngeal nerve injury, hypoparathyroidism, etc.) and permitted the avoidance of thyroid hormone replacement [28,29]. 

Thyroid lobectomy alone may be sufficient initial therapy for low-risk, unifocal, intrathyroidal papillary microcarcinomas (<1 cm) (Figure 1) in the absence of prior head and neck irradiation or clinically involved cervical nodal metastases [4]. The latest 2015 American Thyroid Association (ATA) guidelines endorsed a new category of very low-risk DTCs in which active follow-up represents a viable management option. Evidence supporting active surveillance originated from a large Japanese observational study [30], and was replicated and confirmed in similar studies in the United States [4,31,32].

### 3.2. Extent of Lymphadenectomy

There is consensus that a therapeutic lymph node dissection should be performed in the affected central, lateral, or both compartments when there is evidence of macroscopic lymph node involvement to provide clearance of the disease (Figure 1) [4,26]. 

While prophylactic lateral compartment lymph node dissection is not recommended in the setting of cN0 DTC, the use of prophylactic central compartment lymph node dissection (pCCND) remains controversial [33,34,35,36,37]. Advocates of routine pCCND claim that this procedure decreases the local recurrence rates, increases DSS, improves the interpretation of post-treatment levels of serum Tg, and aids postoperative decision-making regarding the use of adjuvant RAI [33,34,36]. However, patients who underwent pCCND and received postoperative RAI more often experienced higher rates of transient hypocalcemia and overall morbidity than those who underwent total thyroidectomy alone [34]. Prophylactic neck dissection may improve regional control for advanced primary tumors (T3–T4, cN0) [4].

### 3.3. Postoperative RAI Therapy

RAI remnant ablation, adjuvant ablative RAI, and RAI therapy after total thyroidectomy remain essential components in the armamentarium for DTC management. RAI ablation is often justified to eliminate residual clusters of normal thyroid tissue, thus ensuring undetectable serum Tg levels and ^131^I whole-body scans. Secondly, RAI is advocated as an adjuvant treatment to improve long-term outcomes by irradiating presumed occult foci of neoplastic cells within the thyroid remnant. Thirdly, RAI therapy can be used with curative or palliative intention in persistent or recurrent disease [6]. 

The estimated risk of recurrence by the 2009 ATA Initial Risk Stratification System and the American Joint Committee on Cancer/Tumor, Node, Metastasis (AJCC/TNM) Staging System’s predicted mortality is usually used to guide whether and what activity of RAI is given as part of initial therapy [4]. Accordingly, the ATA guideline recommends RAI remnant ablation in low-risk DTC patients only in the presence of specific individual features or other adverse features. RAI adjuvant therapy is considered in ATA intermediate-risk patients and is routinely recommended in high-risk patients after total thyroidectomy [4]. Treatment of a known disease with RAI is regularly recommended in ATA high-risk DTC (Figure 1) [4].

The recently published joint statement by ATA, the European Association of Nuclear Medicine (EANM), the Society of Nuclear Medicine and Molecular Imaging (SNMMI), and the European Thyroid Association (ETA) [38] acknowledged the assessment of the response to initial therapy as an essential step towards the optimization of patient selection for ^131^I therapy, regardless of initial risk stratification [38]. Accordingly, patients with structural, biochemical, or functional evidence of persistent disease can only be candidates for RAI therapy [38]. Patients with no histological, biochemical, or imaging evidence of persistent disease can be eligible for surveillance, remnant ablation, or adjuvant therapy [38].

This joint statement also advises considering multiple factors beyond post-operative disease status and risk stratification, such as patients’ preference and health-care setting in the optimal selection of patients for adjuvant RAI therapy [38]. 

Low activities are usually favored for remnant ablation (30 mCi), while high activities are used for adjuvant (<150 mCi) and treatment purposes [4,6].

To enhance isotope uptake in tumor tissue, ^131^I should be given after TSH stimulation (>30 mU/L), which can be accomplished after thyroid hormone therapy withdrawal for 4–5 weeks [4,6]. The recombinant human TSH (rhTSH) offers a safe and reliable alternative to levothyroxine (LT4) withdrawal in a patient’s preparation for ^131^I therapy. Besides comparable ablation rates, rhTSH has the benefit of avoiding hypothyroidism, lowering radiation exposure, and shortening the duration of hospitalization [39,40,41]. However, thyroid hormone therapy withdrawal is preferred in patients with distant metastases [41].

### 3.4. TSH Suppression following Initial Therapy

TSH-suppressive doses of thyroid hormone therapy have traditionally been used after surgery as a serum to decrease the recurrence risk, as TSH stimulates the proliferation of normal and malignant thyrocytes [18]. 

The TSH suppression in the period immediately following initial therapy is adjusted according to the estimated risk of recurrence, type of surgery, whether the patient underwent remnant ablation RAI, and Tg level. Decision-making at the individual level about the target must also balance the potential benefit of TSH suppression with possible adverse effects from subclinical thyrotoxicosis [42]. 

In the long-term follow-up of DTC, the degree of TSH suppression is chosen according to the ongoing risk stratification. In patients with an incomplete structural response to therapy, ATA offers a solid recommendation to maintain the TSH below 0.1 mU/L in the absence of contraindications (Figure 1). In low-risk DTCs, the TSH may also be kept within the low reference range (0.5–2 mU/L), as long as DFS in these patients was non-inferior to patients with TSH suppression [43]. The evidence supporting an appropriate degree of TSH suppression in high-risk individuals with excellent or indeterminate response to initial therapy is conflicting [44,45,46], and guidelines recommend achieving a target TSH interval of 0.1–0.5 mU/L for up to 5 years in this subset of patients [4]. Based on weak data and experts’ position, ATA guidelines recommend a target TSH of 0.1–0.5 mIU/L in cases with an incomplete biochemical response, meanwhile acknowledging the need for a less or more intense TSH suppression based on initial risk classification, level, and trend of Tg [4].

## 4. Treatment of RAI-Avid Metastatic Disease

Up to 10% of DTCs show distant metastases, raising the overall mortality at 5 and 10 years after diagnosis to 65% and 75%, respectively [47]. Metastases may be identified at initial staging or during long-term follow-up. They are associated with aggressive histological variants, vascular invasion, large primary tumors, macroscopic ETE, bulky locoregional lymph nodes [4,6]. The most common sites of distant metastases are lungs and bones, seen in 49% and 25% of cases, respectively [8]. 

**Figure 2:**
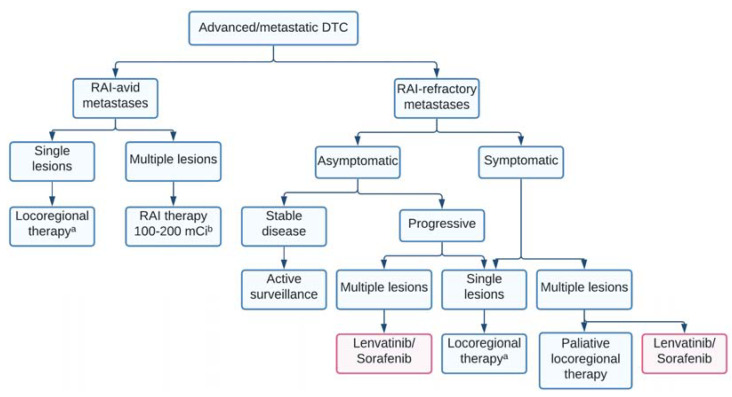
Treatment approach in advanced/metastatic differentiated thyroid cancer. The chart was created using www.lucidchart.com, accessed on 23 January 2022. ^a^ Surgery, External beam radiation therapy or both, radiofrequency ablation, cryotherapy; ^b^ Every six months, for two years, and less frequently after that. Abbreviations: DTC = differentiated thyroid cancer; RAI = radioactive iodine.

Figure 2 illustrates the flowchart of advanced and metastatic DTC management based on metastases’ RAI avidity. The preferred initial treatment for solitary bone lesions in patients with a good performance status is metastasectomy [4]. Following surgery, external beam radiation therapy (EBRT) is associated with the most favorable outcome, especially for limb metastases [48]. RAI therapy may temporarily control the disease and alleviate the symptoms in RAI-avid bone metastases [6]. Bisphosphonates or denosumab have been proven to decrease the risk of skeletal-related events and should be considered in patients with multiple bone metastases.

Radiofrequency ablation (RFA) is a therapeutic alternative for lung oligo-metastases with a diameter less than 2–3 cm in patients not eligible for surgery [6]. 

Patients with RAI-responsive distant metastases may receive 100–200 mCi of ^131^I after TSH stimulation by LT4 withdrawal or rhTSH (Figure 2) [6,8]. The administration of ^131^I is completed every six months for two years and less frequently after that. Between treatments, suppressive doses of LT4 are given to maintain serum TSH levels below 0.1 mIU/mL, unless there are specific contraindications [6,43]. Other directed treatment options such as thermal ablation or external beam radiation therapy may be used as alternatives [4]. 

The validation of two TKIs for DTC, lenvatinib and sorafenib, has led to significant progress in treating advanced TC. Unfortunately, these drugs are not curative, and patients with DTC should have RAIR disease before systemic treatments are considered.

## 5. Treatment of Structural Neck Recurrence

Disease recurrence is defined as a biochemical or structural identification of disease in a patient previously thought to have no evidence of disease. A patient who shows structural, biochemical disease, or both, before being classified as having no evidence of disease is considered to have a persistent disease [49].

The decision regarding surgery versus active surveillance after detecting recurrent or persistent tumors is complex. In the first place, structural, not biochemical, recurrence is required for the decision of surgical approach [50]. Factors to be considered are adverse histology, the magnitude of serum Tg elevation, the rate of lymph nodes growth, worrisome molecular markers, age, comorbidities, patient motivation, and emotional concerns [50,51]. 

Classic revision lateral compartment node dissection (LCND) comprises II, III, and IV levels, while revision central neck dissection includes at least one paratracheal region with prelaryngeal and pretracheal levels [4]. The revision surgery of recurrent DTC poses higher risks of complications than the initial intervention, including vocal fold paralysis, transient or permanent hypoparathyroidism, and injury to major neural structures [50,51]. 

A few minimally invasive techniques, including percutaneous ethanol injection, percutaneous laser ablation and RFA, have been proposed as an alternative to surgery in structural neck recurrence [50]. The main critique of these techniques is that they destroy the lymph nodes in a “berry picking” manner, thus, carrying a higher risk of recurrence [50]. Secondly, they may show distressing local adverse effects, such as inflammation and damage to surrounding structures [50].

## 6. Radioiodine-Refractory DTC

### 6.1. Definition and Current Management

Overall, RAI therapy would be effective in one-third of patients that develop distant metastases. The other two-thirds will, at some point, be classified as RAIR TCs [8]. The basis of RAI therapy is the ability of thyroid follicular cells to capture ^131^I similarly to iodine. NIS is a membrane glycoprotein that facilitates active iodide transport into the cytoplasm of thyroid follicular cells [52]. Similarly, NIS mediates the inclusion of RAI in the cytosol of the cells where it emits beta particles, disrupting tumoral cells [46,47]. In a subgroup of DTCs, the decrease in NIS density on the TC cells’ membrane represents the cause of RAI refractoriness. Thus, NIS is the mainstay of RAIR because NIS loss induces RAI resistance. The prognosis in these cases will vary, depending on the tumor loading and growth rate. MAPK pathway is a central pivot in expressing thyroid-specific genes, including NIS. Consequently, the signaling proteins of the MAPK pathway represent new targets for redifferentiation and, thus, potential therapeutic targets [49]. 

In scientific literature and guidelines [4,53,54,55], identifying patients with RAIR-DTC seems complicated due to the lack of consensus on the definition of RAIR. However, both the ATA guide and most other publications consider the following criteria: (1) the absence of RAI uptake in all lesions on the first therapeutic whole-body scan; (2) cancerous cells become unable to capture RAI after successful treatment courses; (3) the lack of RAI uptake in some but not all lesions; (4) metastatic disease progression despite the appropriate concentration of RAI; (5) reaching the maximum recommended activity of RAI of 22.2 GBq (600mCi).

The development of RAI resistance, which occurs later in the course of the DTC, generally after the initial treatment with RAI, appears especially in patients with multiple, extensive metastases [56]. It seems that the initial RAI therapy leads to the survival of poorly differentiated cells with an inherent resistance to RAI. The probability of progression of these lesions remains higher, mainly when the cells can concentrate 18F-fluoro-2-deoxy-d-glucose (18FDG) [57].

A careful analysis of several clinical features such as age, general health conditions, characteristics of the lesions (e.g., number, size, site, and rate of growth) should be performed before initiating systemic therapy with multi-target kinase inhibitors (MKIs), given the impact of these drugs on patients’ quality of life. Consequently, local therapies, including surgery, are preferred to systemic therapies until the disease progresses and multiple metastases develop [7]. In addition to surgery, the most common types of local treatments are external beam radiotherapy, ablative laser treatment, percutaneous interventional techniques (trans-arterial embolization, TACE), ultrasound (US)-guided ethanol injection, and radiofrequency thermo-ablation [7]. 

### 6.2. First-Line Tyrosine Kinase Inhibitors in Advanced, Metastatic RAIR-DTC

As there are currently no curative treatments for RAIR-DTC, and side effects often accompany MKIs, experts agree that MKIs would be better suited for progressive RAIR disease when local treatment is not an option and when refraining from therapy with MKIs would lead to considerable harm within the near future [26,54,58]. This therapy should be maintained until the disease progresses or adverse effects render it intolerable [58]. 

Table 1 presents the targeted kinase inhibitors that have been tested in randomized-controlled trials (RCTs) for use in advanced, metastatic RAIR-DTC. Lenvatinib and sorafenib are the first-line treatment in RAIR-DTC [6]. Both drugs have been authorised by the European Medicines Agency (EMA) and the United States Food and Drug Administration (FDA) for progressive, metastatic RAIR-DTC. They have been investigated in large, randomized phase III trials, DECISION [59] and SELECT [60], respectively. Head-to-head comparisons of the two agents have not been undertaken. They cannot be compared based on their performances in the RCTs cited above, which differed substantially in terms of enrolment criteria and disease severity.

The DECISION trial included 417 patients treated either with sorafenib (800 mg daily) or placebo, with crossover permitted at disease progression [59]. The study showed that sorafenib significantly prolongs progression-free survival (PFS) (10.8 versus 5.8 months with placebo). Objective responses occurred in 12% of the sorafenib group and 0.5% of placebo-treated patients, and the median response duration was 10.2 months. Stable disease, lasting six months, was observed more frequently with sorafenib (41.8% versus 33.2% with placebo). Disease control was achieved in 54.1% of patients treated with sorafenib and 33.8% receiving placebo. However, the two arms had similar OS.

Donafenib, a derivative of sorafenib, is a novel oral small-molecule presenting complex multiple tyrosine kinase inhibiting mechanisms of action by blocking vascular endothelial growth factor receptor (VEGFR), Raf kinases, and platelet-derived growth factor receptor (PDGFR), thus blocking both angiogenesis and tumor cell proliferation [80]. Donafenib may present improved molecular stability and enhanced pharmacokinetic features compared to sorafenib. Preclinical, phase Ia, and Ib trials have shown favorable efficacy and safety in treating hepatic metastases. A phase 2 trial was conducted recently evaluating the efficacy and safety of two donafenib regimens in Chinese patients with locally advanced or metastatic RAIR-DTC. Both regimens with 200 mg twice daily and 300 mg daily were well tolerated and demonstrated good comparable efficacy in terms of objective response rate [81]. Considering these promising results of dorafenib, a phase III study (NCT03602495) was initiated to determine its optimal dosage and safety using the other two dosage regimens (200 mg versus 300 mg twice daily).

The SELECT trial included 392 patients randomly assigned to either lenvatinib or placebo [60]. The study showed that using lenvatinib as first-line therapy significantly prolonged PFS compared with placebo (PFS 18.3 versus 3.6 months in the placebo arm). A favorable response to lenvatinib was reported in 64.8% of patients (compared with 1.5% in the placebo group) and occurred rapidly (median time to objective response: 2 months). Lenvatinib also significantly reduces tumour burden compared with placebo. The tumour size reduction appeared to occur in 2 phases: rapid diminution observed in the initial period of 8 weeks, followed by slower, continuous shrinkage, with an average rate of −1.3% per month. The drug’s activity varied with the disease site, with lung, hepatic, bone, and lymph node lesions responding promptly. A decrease in Tg levels was also noticed in parallel with the lowering in tumour size, with the maximum decrease achieved at 88 weeks [60]. Unlike placebo, adverse effects were significantly more pronounced with levantinib and were managed by adjusting the dose and medical therapy. [82]. 

Drug-induced adverse effects are commonly noticed. Because these targeted therapies are generally administered long-term, they impact on patients’ quality of life and increase mortality in patients with RAIR-DTC [58]. Although the intended targeted chemotherapy generally induces similar side effects, the use of lenvatinib leads to a higher prevalence of hypertension, and the use of sorafenib leads to a more frequent occurrence of hand-foot syndrome (HFS). Therefore, the use of these two drugs in the initiation of targeted chemotherapy should be personalized, taking into account the patient’s general health, the concurrent existence of other diseases, and the use of other drugs [58]. 

Other antiangiogenic drugs such as sunitinib, vandetanib, axitinib, cabozantinib, pazopanib, and motesanib have been evaluated as first-line therapy in phase II trials, with widely varying RR. Still, none of them has been approved yet for RAIR-DTC (Table 1). 

In a recent report of the primary objective RR analysis and a concurrent preplanned interim PFS analysis of cabozantinib in RAIR-DTC patients (COSMIC-311 trial), the results showed that cabozantinib significantly expanded the PFS (5.7 months versus 1.9 months in the placebo group) and showed a RR of 15% compared to placebo [71]. Cabozantinib also presented adverse effects; the most important ones are palmar-plantar erythrodysesthesia, hypertension, and fatigue. These serious adverse effects occurred in 16% of patients, but there was no treatment-related death [71].

Apart from RCTs, several MKIs have also been evaluated in real-world settings in RAIR-DTC [83,84,85]. In a retrospective study including 101 patients, lenvatinib and pazopanib displayed comparable efficacy to sorafenib. However, serious complications such as hemorrhage, acute coronary syndrome, and thromboembolism occurred more frequently in patients treated with lenvatinib (21%) and pazopanib (13%) compared to sorafenib (7%) [83]. As doses of 24 mg/day of lenvatinib for the treatment of RAIR-DTC presented with high rates of dose reduction, interruption, and discontinuation [85], Jiang et al. investigated the efficacy of a low-dose lenvatinib (median 10 mg/day). The treatment dose achieved acceptable efficacy and outcomes, with a 48-months PFS of 35.6% (95% CI: 18.5–68.4) and 48-months OS of 54.3% (95% CI: 41.2–71.7). In addition, the drug discontinuation rate was only 3.1% [84]. 

Prognostic factors of better outcome with MKIs therapy were identified, such as the absence of 18FDG uptake on target lesions, lower maximum standardized uptake values on positron emission tomography (PET-CT), presence of lung-only metastasis, and lower Tg during treatment [86]. In lenvantinib-treated patients, the appearance of HFS was correlated with a good prognosis, as opposed to fistula formation or severe tumour regrowth after drug discontinuation, which were associated with poor prognosis [87].

In patients with the RAIR-DTC who were positive for one mutation in the genes encoding *BRAFV600E*, *MEK1/2*, or *mTOR* proteins, the efficacy and safety of single targeted kinase inhibitors have been assessed [75,76,77,78,79]. Vemurafenib and dabrafenib are two selective *BRAFV600E* kinase inhibitors currently being evaluated for patients with RAIR-DTC.

The efficacy of vemurafenib in RAIR-DTC patients was not well established due to patient scarcity, and the safety profile displayed a high incidence of serious adverse effects (62%) [75]. The efficacy of dabrafenib was tested in a clinical trial (phase II) including 53 RAIR-PTC patients, randomized to either dabrafenib or dabrafenib combined with trametinib [76]. The study revealed similar high objective response rates with durable responses of 15.6- and 13.3-months median duration response in the two groups [76]. 

Everolimus efficacy and safety were evaluated in a prospective clinical trial (phase II) that enrolled 28 patients diagnosed with RAIR-DTC [79]. During this time, 65% of patients displayed stable disease (SD), with a median follow-up of 38 months and no patients presenting complete or partial response. OS was 18 months, with a PFS of 9 months [79]. Another phase II trial evaluated the genomic profile of tumour cells and found a correlation between the response of RAIR-DTCs to everolimus and the presence of PI3K/AKT mutations [78].

Moreover, currently, a clinical trial (NCT01270321) is evaluating the effectiveness of monotherapy with everolimus (10 mg daily continuously) or pasireotide (1200 mcg twice a day for four weeks followed by long-acting release form, 60 mg i.m. once every four weeks), or their combination in patients with RAIR-DTC and medullary TC (MTC). The trial is currently closed to accrual, and results are expected soon.

### 6.3. Salvage Targeted Therapy after First-Line Tyrosine Kinase Inhibitors Failure

**Figure 3:**
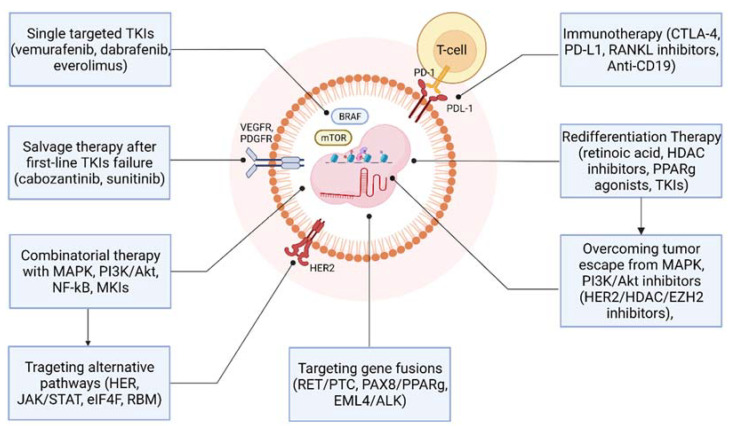
Novel therapeutic approaches in advanced/metastatic RAIR-DTC as alternatives or after first-line MKIs failure. (Created with https://biorender.com/, accessed on 22 January 2022). Abbreviations: eIF4F = eukaryotic initiation factor 4F; EML4/ALK = echinoderm microtubule-associated protein-like 4/anaplastic lymphoma kinase; EZH2 = enhancer of zeste homolog 2; HDAC = histone deacetylase; HER = The human epidermal growth factor receptor; JAK/STAT = Janus Kinase/signal transducer and activator of transcription; *MAPK* = mitogen-activated protein kinase; NF-κB = Nuclear Factor kappa-light-chain-enhancer of activated B cells; PAX8/PPARg = Paired-box gene 8/peroxisome proliferator-activated receptor; PI3K/Akt = phosphatidylinositol-3 kinase/protein-kinase B; RBM = RNA-binding motifs; TKI = tyrosine kinase inhibitor.

Salvage therapy (ST) could be considered both an alternative and an opportunity in clinical practice when there is no benefit under the standard first-line TKIs and when responses are not durable, leading to disease progression or to patients experiencing significant drug toxicity, hardly tolerating the initial TKIs therapy. Figure 3 illustrates an overview of therapeutic approaches addressed in preclinical and clinical studies as alternatives for first-line TKIs, lenvatinib, sorafenib, or after their failure. 

Several trials of TKIs for the treatment of DTC, including patients who received prior treatment with other TKIs, showed that second-line drugs that have similar mechanisms of action might be beneficial after first-line TKIs failure. However, whether the analogies of drug targets in DTC conducted to complete cross-resistance, or whether sequential administration was effective is not entirely known. According to several cases of DTC, clinical cross-resistance of TKIs may not be complete, and objective clinical results were noted when sunitinib or cabozantinib was used as a second agent after sorafenib failure [69]. A phase I trial showed that patients continued to respond to cabozantinib after the previous treatment with sorafenib failed. Five patients obtained partial response (PR) with cabozantinib out of eight patients who received sorafenib, an oral VEGFR-targeted therapy, as first-line therapy [88]. Two patients discontinued sorafenib due to drug toxicity in a phase II trial. Then, one of them followed sunitinib for 12 months and had a 29% reduction in tumour size [64]. 

In patients with metastatic DTC for whom sorafenib failed, other molecular targeted agents were effective as ST after sorafenib failure, despite similar mechanisms of action. In a retrospective study on 60 metastatic RAIR-DTC patients, the median OS of patients receiving ST with sunitinib, pazopanib, cabozantinib, lenvatinib, or vemurafenib was significantly longer compared with sorafenib alone (58 vs. 28 months, *p* = 0.013). The results also indicated that 7 of 17 (41%) patients showed PR, and 10 of 17 (59%) patients had stable disease (SD) with ST. Moreover, the median PFS was 7.4 months with first-line sorafenib and 11.4 months with ST [89]. In a phase II trial that included 22 BRAF-mutated DTC patients treated with vemurafenib, after previously being treated with VEGFR-targeted MKIs (cohort 2), the results have shown benefits such as PR of 27% and median PFS of 8.9 months (95% CI, 5.5 months to not reached), and the median OS of 14.4 months (95% CI, 8.2 to 29.5 months) [75].

A multicenter phase II International Thyroid Oncology Group trial enrolled 25 RAIR-DTC patients with disease progression on prior VEGFR-targeted therapy and evaluated cabozantinib 60 mg/day (or 80 mg/day, if 60 mg/day not effective) for efficacy and safety. Of the 25 patients, 40% presented with a PR, 52% had stable disease, and 8% were not evaluable. Cabozantinib demonstrated relevant efficacy with a median PFS of 12.7 months and an OS of 34.7 months. The most frequent adverse effects were diarrhea, fatigue, palmar-plantar erythrodysesthesia, weight loss, and hypertension [69]. In an ongoing phase III clinical trial (NCT03690388), investigators are also evaluating the efficacy of cabozantinib versus placebo in 258 subjects with RAIR-DTC, for whom the disease has progressed after prior treatment with a VEGFR inhibitor. Results are expected at the end of 2022. The mechanisms by which second-line cabozantinib may induce clinical benefit are not fully established, although it may be related to c-MET (transmembrane tyrosine kinase that binds the hepatic growth factor) resistance induced by previous VEGFR therapy [69]. Because FTCs or PTC cases harboring *NRAS* or *KRAS* mutations exhibit the maximum tumour shrinkage, the tumour genotype may represent a predictor of response to cabozantinib.

## 7. Combinatorial Therapy with MAPK, PI3K/Akt/mTOR, NF-κB, and MTKs Inhibitors

It has become a highly advocated therapeutic strategy to simultaneously point at more targets using drug combinations for the treatment of TC. This strategy would likely improve the low therapeutic efficiency achieved with single-agent treatments in clinical trials on cancer, including TC.

The most studied combinations target the MAPK pathway with PI3K/Akt/mTOR pathway inhibitors, MTKIs, or Nuclear Factor kappa-light-chain-enhancer of activated B cells (NF-κB) pathway inhibitors. PI3K/Akt/mTOR signaling constitutes an important pathway consisting of phosphatidylinositol 3-kinase (PI3K) and serine/threonine-protein kinase B (AKT). Other therapies use MTKIs with PI3K/AKT/mTOR inhibitors to increase treatment efficacy (Table 2).

A study of TC preclinical models evaluated a novel drug combination that inhibited PI3K/AKT/mTOR and RAF/MEK/ERK pathways and was active against VEGFR2. Dactolisib (BEZ235), a PI3K inhibitor, and RAF265, a RAF inhibitor active against VEGFR2, were tested, and both drugs considerably inhibited their kinase targets and strongly inhibited proliferation of DTC (but also MTC) cell lines with mutations in *RAS*, *BRAF*, ret proto-oncogene (*RET*), and *PTEN* [90]. Some of the explanations that were found underlie this synergy and include downstream convergence of translational control of survival signaling [96] and regulation of the balance of pro-apoptotic and anti-apoptotic members of the Bcl-2 family (e.g., BIM up-regulation, a pro-apoptotic BH3 protein) [97].

Another preclinical study tested the effects of the MEK inhibitor, refametinib (RDEA119), and the mTOR inhibitor, temsirolimus, on TC cells and proved that their combined utilization had a considerable synergism in inhibiting the proliferation of TC cells in vitro and the growth of xenograft thyroid tumors, demonstrating a promising therapeutic potential. The impact of the combination therapy using RDEA119 and temsirolimus on cell proliferation or autophagic death was more pronounced in cells that harbored genetic alterations in the MAPK and PI3K/AKT/mTOR pathways [98]. Besides, MK2206, an allosteric AKT-specific inhibitor, profoundly synergized with vemurafenib (BRAFV600E inhibitor) or selumetinib (MEK-1/-2 inhibitor) in inhibiting TC cells, harboring activating mutations in both the PI3K/AKT/mTOR and MAPK pathways. This synergism was absent or weak in TC cells harboring single or no mutations in the two pathways [92].

In vitro and in vivo studies with combined therapy with PLX4720 (BRAF inhibitor) and ponatinib (MTKI) also reported optimal anticancer results on *BRAFV600E* positive cells [93]. This combination appears to have helped the cells surmount the resistance to PLX4720, as Ghosh et al. demonstrated [94]. 

The evidence of a preclinical study also suggests that a combinatorial approach that inhibits both the MAPK and PI3K/AKT/mTOR pathways, by using sorafenib and dactolisib, exerts a more significant antitumor effect than sorafenib alone in TC cell lines [91]. 

The MAPK pathway interacts with the PI3K/Akt/mTOR pathway and the NFκB cascade, indirectly targeted by the bortezomib. The interaction between the agents targeting these pathways, vemurafenib, and bortezomib, resulted in a synergistic antiproliferative effect in *BRAFV600E*-bearing PTC cells and xenograft models [95].

## 8. Targeting Alternative Pathways to Overcome the Resistance to MAPK and PI3K Pathways Inhibitors

One critical aspect of improving cancer therapy is to inhibit the primary oncogenic pathway and simultaneously prevent functional redundancies and pathways crosstalk that facilitates survival of malignant cells, rendering tumors resistant to treatment [94]. Different compensatory mechanisms have been shown to promote this resistance bypassing pharmacologic inhibition of BRAFV600E via the triggering of intracellular signaling cascade pathways, leading to reactivation of phospho(p)—extracellular signal-regulated kinase 1/2 (ERK1/2). 

The human epidermal growth factor receptor 3 (*HER3*) overexpression in TC cells presenting the *BRAFV600E* mutation leads to aberrant autocrine loops. The overexpression of the HER signaling pathway results in reactivation of the extracellular signal-regulated kinase (ERK), AKT, or both pathways, in the context of BRAF or MEK inhibitors treatment [99]. HER2 and HER3 receptors stimulate and trigger the activation of the Janus kinase (JAK)-signal transducer and activator of transcription (STAT) pathways and the SRC kinase, leading to treatment failure [100] (Figure 3).

Other mechanisms are related to eukaryotic initiation factor 4F (eIF4F) complex formation, which is implicated in the reactivation of ERK1/2 signaling or persistent activation of phosphorylated-mTOR and phospho-S6 ribosomal proteins signaling [101]. Combinations of drugs targeting BRAF (and/or MEK) and eIF4F, such as vemurafenib combined with the flavagline FL3, silvestrol, or hippuristanol (a potent eIF4A inhibitor), may overcome most of the resistance mechanisms arising in the BRAFV600-mutant cancers [101]. 

Resistance to the BRAFV600E inhibitors is also mediated by dimerization of aberrantly spliced BRAFV600E that dimerize in a RAS-independent manner [102]. *NRAS* mutations, *BRAFV600E* amplifications, *MEK1/2* mutations, and overexpression of genes including tumor progression locus 2 (*COT*), *PDGFR-B*, and induced myeloid leukemia cell differentiation protein (*MCL1*) copy number gain, play a role in the resistance to BRAFV600E inhibitors [103].

Vemurafenib-resistant cells show amplification of chromosome 5 and de novo mutations in the RNA-binding motifs (*RBM*) genes family (e.g., *RBM 10*, *RBMX*). *RBMX* knockdown in naïve-cells contributes to tetraploidization, including expansion of clones with chromosome 5 aberrations [104]. *RBMX* elicits gene regulatory networks with chromosome 5q cancer-associated genes and pathways for G2-M and DNA damage-response checkpoint regulation in *BRAFWT/V600E*-PTC. Combinatorial treatment with vemurafenib plus palbociclib (inhibitor of CDK4/6, mimicking P16 functions) synergistically induces more substantial apoptosis than monotherapy in vemurafenib resistant-cells of the TC harboring *BRAFWT/V600E* mutation [104].

## 9. Redifferentiation Therapy

### 9.1. Redifferentiation Therapy Achievements Acquired during Recent Decades

Although in recent years significant success has been achieved by using MKIs, there are still not many therapeutic options for patients with RAIR-DTC. Because RAI resistance originates in DTC loss of differentiation, treatment for redifferentiation, and subsequent RAI therapy, would represent an advisable treatment strategy for RAIR-DTC patients. Figure 2 presents the drugs currently used or tested for redifferentiation therapy and their molecular targets. 

Based on encouraging results in preclinical studies, such as in vitro redifferentiation of thyroid carcinoma cells by increasing NIS, type 1 iodothyronine deiodinase, and alkaline phosphatase, generating an increase in ^131^I uptake and TSH binding, retinoic acid has a long history of clinical evaluation. However, clinical results were not conclusive, primarily because of the patients’ heterogeneity [105]. A recent retrospective study showed an improvement in ^131^I uptake using retinoic acid [105]. Nonetheless, the evaluation of BRAF mutational status before redifferentiation therapy could help predict response and choose the appropriate redifferentiation therapy, as 67% of patients with *BRAFV600E* mutation benefited from RAI therapy in comparison to 56% of patients without BRAF mutation after treatment with retinoic acid after 2.5-year follow-up, suggesting that the evaluation of BRAF mutational status prior to redifferentiation therapy could be useful for predicting response and choosing the appropriate redifferentiation therapy [48]. 

Retinoids, as opposed to TKIs, have fewer side effect; however, due to the low effectiveness the clinical interest in retinoic acid remains limited [48].

Previous in vitro studies have shown that histone deacetylase (HDAC) inhibitors (e.g., valproic acid, vorinostat, romidepsin, belinostat, panobinostat) have promising effects in cancer therapy by inhibiting tumor cell proliferation, inducing apoptosis, cell cycle arrest, and differentiation in the brain, hematological and TCs [106,107,108]. Upon authorization of vorinostat by the FDA to treat T-cell lymphoma, an additional investigation was conducted on 19 patients diagnosed with TC [109]. The study was terminated due to progression of the disease by Response Evaluation Criteria in Solid Tumors (RECIST) criteria (*n* = 7), clinical progression (*n* = 3), and grade 1–3 AE (fatigue, dehydration, ataxia, pneumonia, bruises, and deep vein thrombosis) [99]. Additionally, based on encouraging in vitro results, romidepsin was further evaluated in 20 patients diagnosed with RAIR-DTC. Although RAI reuptake occurred in 2 patients and 13 patients presented stable disease, the inability to meet RECIST criteria and the documentation of two grade 4–5 adverse effects (sudden death and pulmonary embolus) inhibited continuation beyond the first stage [110]. Thus, as monotherapy, HDAC inhibitors have not proven major clinical benefits in RAIR-DTC patients. 

Rosiglitazone is a peroxisome proliferator-activated receptor (PPAR) gamma agonist that has shown promise in preclinical studies as both an antiproliferative and redifferentiation agent for TC treatment [48,106,111]. However, a clinical trial (phase II) suggests that rosiglitazone therapy may induce RAI uptake and decreased plasma Tg levels in some patients with DTC, without a clinically significant response on long-term follow-up. Moreover, no response to rosiglitazone therapy was observed in imaging studies [107].

### 9.2. Modulation of MAPK and PI3K/AKT/mTOR Pathways in Restoring DTC Radio-Sensitivity: Preclinical and Clinical Evidence

**Figure 4:**
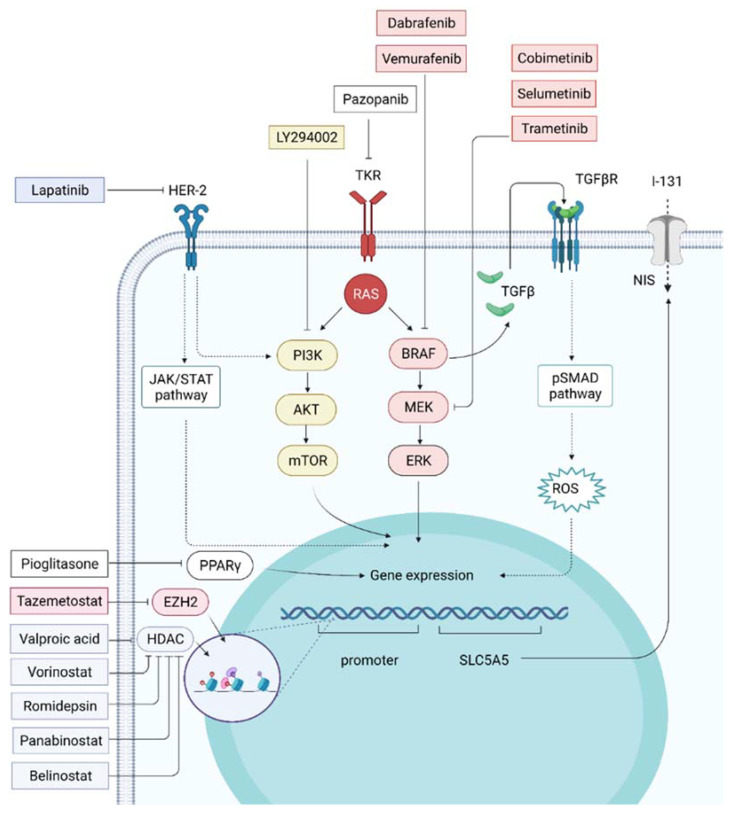
Molecular targets of drugs used in redifferentiation therapy (Created with https://biorender.com/, accessed on 22 January 2022). BRAF inhibitors—dabrafenib, vemurafenib; MEK inhibitors—cobimetinib, selumetinib, trametinib; PI3K inhibitor—LY294002; multitargeted tyrosine kinase receptor blocker—pazopanib; HER2 inhibitor—lapatinib; PPAR-γ inhibitor—pioglitazone; EZH2 inhibitor—tazemetostat; HDAC inhibitors—valproic acid, vorinostat, romidepsin, panobinostat, belinostat.

Recent studies that evaluated drugs targeting MAPK and PI3K presented contradictory results about RAI therapy efficacy in RAIR-DTC. Figure 4 illustrates the previously and recently studied drugs in redifferentiation therapy of RAIR-DTC and their molecular targets.

Selumetinib (MEK1/2 inhibitor) was used as the first MAPK inhibitor to restore ^131^I uptake in RAIR-DTC patients. Based on encouraging initial results and a favorable safety profile from the phase II study [112,113], a multicentric phase III RCT was launched to evaluate whether adding selumetinib to adjuvant RAI could improve clinical practice outcomes in RAIR-DTC patients. However, Ho et al. have shown no significant difference between selumetinib and the placebo groups regarding complete remission rate [114]. 

Patients with RAIR PTCs and *BRAFV600E* mutation achieved ^131^I re-uptake after treatment with dabrafenib, a selective inhibitor of mutant BRAF, thus achieving redifferentiation in terms of clinical, biochemical, metabolic, and histological effects [115]. Even so, treatment results showed that NIS expression recovery does not guarantee restoration of radio-sensitivity [115].

Vemurafenib, by contrast to dabrafenib, induced restoration of radio-sensitivity after six months of treatment in almost 20% of patients. However, the heterogeneity of this effect makes it difficult to evaluate it in RAI non-responders [116].

It has been hypothesized that pazopanib might synergize with ^131^I by improving RAI delivery, thus enhancing ^131^I efficacy. A phase I clinical trial using pazopanib at standard fixed doses combined with increasing doses of ^131^I showed that pazopanib is not an attractive option for restoring radiosensitivity due to both a low efficacy (no improvement in RAI uptake) and increased toxicity (cardiovascular and hematologic) [117].

Dual targeted inhibition of BRAF and MEK by dabrafenib and trametinib, respectively, increased NIS expression in patient-derived, RAI naïve-PTC cell cultures more efficiently than trametinib in monotherapy [118]. A recent case report presented the successful redifferentiation of a previously RAI-refractory follicular TC after treatment with a dabrafenib and trametinib combination in a patient who could not tolerate MKIs toxicity [119]. 

Preliminary results from an ongoing phase II clinical trial (NCT01723202) show similar tolerability for the single-agent dabrafenib and the dabrafenib-trametinib combination [76]. In addition, in clinical settings, a significant number of patients who regained ^131^I sensitivity presented with a clinical response, mainly when a higher ^131^I uptake was associated with high serum Tg levels [120].

These preliminary results are currently being evaluated in a phase II clinical trial (NCT03244956) using a combined therapy (trametinib plus dabrafenib) in patients diagnosed with RAIR-DTC with either *RAS* or *BRAFV600E* mutations. However, no results have been presented yet, as the study is estimated to be completed in December 2022. Table 3 presents the clinical studies evaluating multi- and single-targeted kinase inhibitors in DTC redifferentiation therapy.

The inhibition of *NIS* expression can result from the PI3K/AKT/mTOR pathway hyperactivation; thus, drugs targeting this pathway could be attractive options in RAIR-DTC [123]. LY294002, a PI3K inhibitor, activates the Paired-box gene 8 (*PAX8*) in TC cells, significantly increasing *NIS* expression and, thus, the uptake of iodide [124,125]. Another PI3K inhibitor, rapamycin, was found to induce NIS protein levels along with a RAI uptake in *BRAFV600E* and *RET/PTC1* PTC derived cell lines, possibly through a transcriptional effect dependent on the transcription factor TTF1 [126]. Rapamycin and its synthetic analogue, everolimus, provided controversial results in a non-tumoral thyroid in vitro model [124,127]. An ongoing phase Ib clinical trial (NCT04462471) is also currently evaluating the efficacy of the combination vemurafenib plus copanlisib (a PI3K inhibitor) for reversing RAIR in patients diagnosed with RAIR-DTC, exhibiting *BRAFV600E* mutations and also establishing the maximum tolerated dose for this combination to minimize the risk of the adverse effects. Hence, further studies are needed to evaluate the efficacy of PI3K/AKT/mTOR pathway inhibitors for the treatment of RAIR-DTC.

### 9.3. Perspectives in Overcoming Tumour Escape from MAPK and PI3K Inhibitors, and RAI Resensitizing Effect

Although TKIs have improved the RAI sensitivity of TC cells, the problem of RAIR-DTC is still concerning, and, even if successful therapy can be achieved, after several months, the resistance can occur. Table 4 presents the preclinical studies evaluating the combination of MAPK pathway inhibitors with HER2/HDAC/EZH2 inhibitors to restore RAI sensitivity.

In vitro studies using BRAF/MEK inhibitors in combination with HER inhibitors have shown redifferentiation affects the BRAFV600E mutant PTC cells compared to BRAF/MEK inhibitors alone. Herein, the HER1/2 inhibitor lapatinib appeared to prevent *MAPK* activation and increased the sensitivity of BRAFV600E mutant PTC cells to dabrafenib or selumetinib (phase I clinical trial is ongoing) [128]. Moreover, the combined inhibition of BRAF and HER3 using vemurafenib and the novel human monoclonal antibody CDX-3379 was shown to provide safety and efficacy in increasing RAI absorption in a pilot clinical trial involving seven patients. The results suggest that mutations in the SWItch/Sucrose Non-Fermentable (*SWI/SNF*) gene AT-Rich Interaction Domain 2 (*ARID2*) should be investigated as potential markers of resistance to redifferentiation strategies [131]. 

The histone H3 lysine 27 (H3K27) trimethylation modification (H3K27me3) decreases the gene expression through the enhancer of zeste homolog 2 (EZH2), a critical methyltransferase catalyzing H3K27, and an epigenetic mark for the maintenance of gene silencing. Hypertrimethylation of H3K27 proved to be associated with tumour cells’ dedifferentiation and resistance to the BRAF inhibitors. MAPK pathway aberrant activation by BRAFV600E in TC also increased the level of H3K27me3 by increasing the expression of *Ezh2*. Therefore, specific inhibition of EZH2 represents a potential direction of differentiation therapy.

In this context, emerging studies have revealed that the EZH2 inhibitor, tazemetostat, in combination with MAPK inhibitors (dabrafenib or selumetinib), promoted ^125^I uptake in *BRAF600E*-mutated PTC cell lines and enhanced both NIS and TSHR expression [64]. These two drugs also inhibited EZH2 activity, yielding a substantial reduction of the downstream H3K27me3. This combination, including MAPK inhibitors and tazemetostat may potentially translate into a novel differentiation therapeutic strategy [129]. Besides, a recent study evaluating patients with resistance to sorafenib RAIR-DTC revealed that EZH2 was the direct target for microRNA (miR)-124 and miR-506 [130]. Furthermore, the sorafenib resistant cells regained sensitivity for sorafenib with miR-124/506 overexpression of EZH2 inhibitors, which led to the decreased trimethylation at H3K27me3. Therefore, suppression of EZH2 combined with sorafenib represents a potential target for TC therapy [130].

## 10. Immunotherapy of Advanced TC

The development of immunotherapeutics and immune checkpoint inhibitors, such as anti-cytotoxic T-lymphocyte-associated protein 4 (CTLA-4) and anti-programed death 1 (PD-1) molecules are being studied in clinical trials for the treatment of different types of cancer [116]. PD-1 ligand 1 (*PD-L1*) overexpression has been documented in DTC [132]. *PD-L1* expression by tumor cells has also been correlated with a higher risk of recurrence and reduced DFS in PTC [116,133]. Thus, targeting these immune system components may also prove helpful in treating RAIR-DTC.

In a phase Ib clinical study (KEYNOTE-028), pembrolizumab (PD-1 inhibitor) presented high tolerability in patients diagnosed with PTC or FTC that had progressed with standard therapy. After a median follow-up of 31 months, two patients (9%) achieved an overall partial response with a response duration of 8 and 20 months, respectively [134]. In 13 other patients, the median duration of stable disease was seven months. Although researchers enrolled only tumors that expressed PDL1, this poor response might be related to the inclusion of heavily pretreated advanced TCs predicted from the start to be resistant to immune checkpoint inhibitors monotherapy. 

Various combinations with immunotherapy drugs are being investigated in ongoing clinical trials (Table 5), including RAIR-DTC patients. A phase II clinical trial (NCT04061980) pointed to assess the efficacy and safety of encorafenib (BRAF inhibitor) and binimetinib (MEK inhibitor) with or without nivolumab (anti-PD-1 antibody) in patients with *BRAF V600*-positive metastatic and RAIR-DTC. Encorafenib, binimetinib, in association with pembrolizumab (anti-PD-1 antibody), were evaluated previously in patients with *BRAFV600E* positive melanoma in the IMMU-TARGET trial [135], with favourable results, thus, encouraging the use of MAPK inhibitors in combination with immunotherapy in other types of cancer, including TC. Another phase II RCT (NCT02973997), combining therapy with pembrolizumab and lenvatinib (VEGFR inhibitor) in lenvatinib-naïve patients with progressive RAIR DTC, is ongoing, and results are expected soon [136].

However, in response to anti-VEGF therapies, some tumors can increase fibroblast growth factor (FGF) secretion, thus increasing endothelial cell proliferation, promoting tumour angiogenesis, and evading VEGF signaling inhibition. It appears that stimulating VEGFR, FGF receptors (FGFRs), and colony-stimulating factor 1 receptors (CSF1R) also promotes tumour immune evasion. VEGF secreted by tumors can activate VEGFR signaling pathways in T cells, leading to PD-1 receptor overexpression and inhibiting T cell anti-tumour activity [137]. In addition, FGFR and CSF1R stimulate tumour-associated macrophage proliferation and differentiation, thus promoting tumour immune evasion [138]. By targeting multiple kinases and simultaneously blocking VEGFR-, FGFR-, and CSF1R-mediated pathways, sulfatinib may prevent tumour angiogenesis and tumour immune evasion [139]. In this respect, an ongoing multi-center, phase II clinical trial (NCT02614495) that included 66 patients diagnosed with advanced MTC and RAIR-DTC aims to evaluate the efficacy and safety of sulfatinib 300 mg.

PD-1 and CTLA-4 immune checkpoint inhibitors such as nivolumab and ipilimumab, promote antitumor immune responses by complementary mechanisms, synergistically affecting different signaling pathways. PD-1 contributes to T-cell scarcity in the tumour microenvironment, and CTLA-4 inhibits activated and regulatory T cells in the lymphoid organs. Nivolumab, associated with ipilimumab, has proved efficacy in the therapy of renal carcinoma, lung cancer, melanoma, metastatic colorectal cancer, and hepatocellular carcinoma [140]. The combination of cabozantinib (VEGFR inhibitor) with nivolumab (anti-PD-1) and ipilimumab (anti-CTLA-4) is currently being investigated in patients with RAIR-DTC in a clinical trial (phase II), including 24 RAIR-DTC patients with disease progression after monotherapy with anti-VEGFR (NCT03914300).

In addition, two clinical trials are currently recruiting patients with RAIR-DTC. The first trial (NCT04560127) is investigating the efficacy of camrelizumab (anti-PD-1 immune checkpoint inhibitor) in association with apatinib (VEGFR2/KDR inhibitor), and the second trial (NCT04544111) is evaluating both efficacy and safety of the association of spartalizumab (PD-1 inhibitor) with trametinib or dabrafenib.

In patients with bone metastatic RAIR-DTC, an ongoing phase II clinical trial (NCT03732495) is currently recruiting patients to evaluate the efficacy of the combination lenvatinib plus denosumab (receptor activator of nuclear factor kappa-B ligand - RANKL inhibitor).

Anti-CD19 chimeric antigen receptor T cell (CAR-T) therapy is a modern type of immunotherapy that has successfully been used to treat numerous cancers, including animal models of TC [117]. Results from preclinical trials have highlighted the effectiveness of CAR-T against TC cell lines [141], raising the possibility of also using it for advanced RAIR-DTC.

## 11. Targeting Gene Fusions in Differentiated Thyroid Cancer

Chromosomal rearrangements result from breakage and fusion within a chromosome or amongst different chromosomes, which leads to various gene alterations, including gene fusions [142]. Kinase gene rearrangements lead most often to dysregulation in intracellular signaling involving essential systems such as RAS/RAF/MAPK, PI3K/AKT/mTOR, or JAK/STAT and are frequently involved in carcinogenesis. Rearrangements of genes encoding tyrosine-kinase receptors lead to ligand-independent dimerization, thus, being oncogenic drivers in many types of cancer, including TC. Actionable kinase gene rearrangements are found in genes coding for anaplastic lymphoma kinase gene (*ALK*), *RET*, *MET*, neurotrophic tyrosine receptor kinase (*NTRK*), fibroblast growth factor receptor (*FGFR*), or ROS proto-oncogene 1 receptor (*ROS1*) [143,144,145].

One of the most common gene fusions in TC is *RET/PTC*, occurring in 10–20% of all PTCs [146]. It results from the merging of *RET* gene segment with a different pair gene, thus leading to excessive activation of MAPK and PI3K/mTOR/AKT pathways [147]. *RET/PTC* generates oncogenic fusion proteins, more frequently in younger patients and associated with DNA damage after ionizing radiation (e.g., up to 60% cases of post-Chernobyl PTCs) [148,149].

In the *PAX8/PPARg* rearrangement, a segment of the PAX8 gene fuses with the peroxisome proliferator-activated receptor-gamma (*PPARg*) gene. According to several studies, this fusion oncogene is present in up to 60% of FTC and FVPTC [150,151,152]. A phase II trial (NCT01655719) described the efficacy of pioglitazone, a PPARg agonist, in a patient with advanced RAIR Hurthle cell carcinoma harboring the *PAX8*/*PPARg* translocation, translated into a significant decrease in metastatic lesion dimensions and Tg level after 24 months of therapy [153].

Another recently identified translocation, Echinoderm microtubule-associated protein-like 4/anaplastic lymphoma kinase *EML4/ALK*, carries potential therapeutic implications in DTC. *EML4/ALK* gene fusions result in pathological *ALK* kinase activation, leading to oncogenic signaling via different pathways, such as PI3K/AKT, RAS/ERK, JAK/STAT [154]. This genetic alteration was identified in a patient with metastatic RAIR-PTC who targeted successively with two ALK TKIs, crizotinib, and lorlatinib. After six months of treatment with crizotinib, the patient had a stable disease. However, cerebral metastases were found after eight months. With lorlatinib, the patient achieved ongoing PR after seven months of therapy. The study showed that lorlatinib was more efficient than the already administered crizotinib in vivo and in vitro [155].

Lately, more selective drugs, such as larotrectinib (anti-TRK), entrectinib (anti-ALK, ROS1, and TRK), selpercatinib, and pralsetinib (both anti-RET), have proved significantly improved benefit–risk balance in clinical trials with kinase fusion-positive thyroid carcinoma patients [145].

## 12. Conclusions and Future Perspectives

Although most DTCs have an excellent prognosis under the conventional treatment (surgery followed by ^131^I therapy and TSH suppression) and present a near-normal life expectancy, in a minority of cases, local or distant metastases can occur, decreasing the survival in this subset of patients. RAI therapy, the mainstay treatment in advanced, metastatic, and recurrent disease may become ineffective at some point in two-thirds of these cancers due to the development of RAI-refractoriness. Recent advances in discovering molecular mechanisms underlying DTC have shifted the focus of TC therapy from a standard approach to those targeting specific genetic dysregulation. The first success in targeted therapy of RAIR-DTC was achieved with the approvement of two first-line MKIs, lenvatinib and sorafenib, by the Food and Drug Administration. However, some RAIR-DTC can escape from the effect of first-line TKI agents after several months. Salvage therapy after first-line TKI failure may be tried out with cabozantinib, sunitinib, pazopanib, nevatinib, or vemurafenib. Other agents are currently being investigated, and combinatorial therapy, immunotherapy, redifferentiation therapy, use of agents targeting alternative pathways are some of the strategies that have been proposed to address this challenge. Combinatorial therapy has been evaluated using MAPK inhibitors associated with PI3K signaling cascade inhibitors, TKIs, or even NF-κB inhibitors. There is high hope for clinical results, although most combinations have delivered beneficial results only in preclinical trials. However, resistance to MAPK and PI3K pathways inhibitors may be a concern. Thus, therapies targeting *HER3*-overexpression, JAK/STAT, *SRC*-kinase activation, eIF4F complex, or CDK4/6 are exciting targets for overcoming this type of resistance. Nevertheless, RAI refractoriness is predominantly due to DTC dedifferentiation, as such redifferentiation therapies using BRAF, MEK, PI3K inhibitors, MTKIs, HER2, PPAR-γ, EZH2, or HDAC inhibitors are currently being analyzed for efficacy.

Moreover, several phase II clinical trials are evaluating the potency of immunotherapies targeting CTLA-4, PD-1, FGFR-1, CSF1R, or RANKL. Lastly, targeting gene rearrangements can be associated with a more favorable benefit–risk balance. Drugs currently under evaluation in kinase fusion-positive thyroid carcinoma patients are the anti-ALK/ROS1 crizotinib and lorlatinib, the anti-TRK larotrectinib, the anti-ALK, -ROS1, -TRK entrectinib, anti-RET selpercatinib, and pralsetinib.

## Figures and Tables

**Table 1:** RCTs results of targeted chemotherapies in RAIR-DTC.

Name of the Drug	Targets	Phase II/III	RCT	PFS (Months)	RR (%)
**Multitargeted Kinase Inhibitors**					
Sorafenib	VEGFR, PDGFR, c-KIT, RET, RAF	III	DECISION [59]	10.8	12
		II	Gupta et al., 2008 [61]	79	23
		II	Kloos et al., 2009 [62]	15	15
Lenvatinib	VEGFR, PDGFR, c-KIT, RET, FGFR	III	SELECT [60]	18.3	64.8
		II	Cabanillas et al., 2015 [63]	12.6	50
Sunitinib	VEGFR, PDGFR, c-KIT, RET, FLT3, GCSFR	II	Carr et al., 2010 [64]	12.8	31
		II	Ravaud et al., 2017 [65]	22	13.1
Vandetanib	VEGFR, EGFR, RET	II	Leboulleux et al., 2012 [66]	11	8.3
Axitinib	VEGFR, PDGFR, c-KIT, RET	II	Cohen et al., 2008 [67]	18	30
		II	Locati et al., 2014 [68]	16	35
Cabozantinib	VEGFR, RET, c-MET, FLT3, TEK	II	Cabanillas et al., 2017 [69]	12.7	40
		II	Brose et al., 2018 [70]	NA	54
		III	Brose et al., 2021 (COSMIC-311) [71]	5.7	15
Pazopanib	VEGFR, PDGFR, c-KIT	II	Bible et al., 2010 [72]	11.7	49
		II	De la Fouchardiere et al., 2021 (PAZOTHYR) [73]	9.2	35.6
Motesanib	VEGFR, PDGFR, c-KIT, RET	II	Sherman et al., 2008 [74]	9.3	14
**Single Targeted Kinase Inhibitors**					
Vemurafenib	BRAFV600E	II	Brose et al., 2016 [75]	15	38.5
Dabrafenib	BRAFV600E	II	Shah et al., 2017 [76]	11.4	50
		I	Falchook et al., 2015 [77]	1.3	29
Everolimus	mTOR	II	Hanna et al., 2018 [78]	12.9	6
		II	Schneider et al., 2016 [79]	9	0

RCT—randomized controlled trial; PFS—progression-free survival; RR—response rate; BRAF—v-raf murine sarcoma viral oncogene homolog B1; c-MET—hepatocyte growth factor receptor or HGFR; c-KIT—stem cell factor receptor or SCFR; EGFR—epidermal growth factor receptor; FGFR—fibroblast growth factor receptor; FLT3—FMS-like tyrosine kinase 3 (or CD135); GCSFR—granulocyte colony-stimulating factor receptor (or CD114); MEK—mitogen-activated protein kinase; mTOR—mammalian target of rapamycin; NA—not available; PDGFR—platelet-derived growth factor receptor; RET—ret proto-oncogene; RAF—rapidly accelerated fibrosarcoma; VEGFR—vascular endothelial growth factor receptor; TEK—non-receptor tyrosine kinase.

**Table 2:** Most studied combinatorial therapies for advanced differentiated thyroid cancer.

Targets		*MAPK* Pathway		Multiple Tyrosine Kinases
		RAF/BRAFV600E	MEK-1/-2	
PI3K/AKT/mTOR pathway	PI3K	RAF 265 + Dactolisib (BEZ 235) (Jin et al., 2011) [90]		Sorafenib + Dactolisib (Yi et al., 2017) [91]
	mTOR		Refametinib + Temsirolimus (Liu et al., 2012) [92]	Sorafenib + Temsirolimus (Sherman et al., 2017) [93]
	AKT	MK 2206 + Vemurafenib (Liu et al., 2012) [92]	MK 2206 + Selumetinib (Liu et al., 2012) [92]	
Multiple tyrosine kinases		PLX4720 + Ponatinib (Ghosh et al., 2020) [94]		
NF-κB		Vemurafenib + Bortezomib (Tsumagari et al., 2018) [95]		

BRAFV600E—BRAF mutation (thymidine to adenosine substitution resulting in the replacement of valine with glutamic acid at amino acid 600 in the 15th exon); BRAF—v-raf murine sarcoma viral oncogene homolog B1; *MAPK*—mitogen-activated protein kinase; MEK—mitogen-activated protein kinase; PI3K—phosphatidylinositol 3-kinase; Akt—serine/threonine-protein kinase B; mTOR—mammalian target of rapamycin.

**Table 3:** Clinical studies evaluating multi- and single-targeted kinase inhibitors in DTC redifferentiation therapy.

Drug (target)	Study Type	Patients (Number)	RAI Uptake Threshold	PR	SD	PFS (Month)	Study
**Monotherapy**							
Selumetinib (MEK-1/-2)	Prospective	20	8/20	5/8	3/8		Ho et al., 2013 [112]
	II	3		1/32	21/32	8	Hayes et al., 2012 [121]
	III	157		60/157			Ho et al., 2018 [114]
Dabrafenib (BRAF)	I Prospective	10	6/10	2/6	4/6		Rothenberg et al., 2015 [115]
Vemurafenib (BRAF)	Pilot Prospective	10	6/10	2/4	2/4	6	Dunn et al., 2019 [109]
	Retrospective	6	4/6	3/4	1/4		Iravani et al., 2019 [122]
Pazopanib (MTKI)	I	6	0/6 *	0/6	5/6	6.7	Chow et al., 2017 [110]
**Combined Therapy**							
Dabrafenib/Verumafenib + Trametinib/Cobimetinib (MEK)	Retrospective	6	4/6	3/4	1/4		Jaber et al., 2018 [120]
Dabrafenib + Trametinib	II	53	-	9/24	10/27	15.1	Shah et al., 2017 [76]

SD—stable disease; PR—partial response; PFS—progression-free survival; BRAF—v-raf murine sarcoma viral oncogene homolog B1; MEK—mitogen-activated protein kinase; MTKI—multitargeted kinase inhibitors; * Insignificant increase.

**Table 4:** Preclinical studies evaluating the combination of *MAPK* pathway inhibitors with HER2/HDAC/EZH2 inhibitors in re-sensitizing RAIR-DTC to RAI therapy.

Combination of *MAPK* Pathway Inhibitors with:	Preclinical Studies Targeting Redifferentiation Therapy of RAIR-DTC	References
HER2 inhibitors	Dabrafenib (BRAF) + lapatinib (HER2)	Cheng et al., 2017 [128]
	Selumetinib (MEK-1/-2) + lapatinib (HER2)	Cheng et al., 2017 [128]
HDAC inhibitor	Dabrafenib/selumetinib + panabinostat (HDAC)	Fu et al., 2020 [129]
EZH2 inhibitor	Dabrafenib/selumetinib + Tazemetostat (EZH2)	Fu et al., 2020 [129]
	Selumetinib + Tazemetostat (EZH2)	Wang et al., 2019 [130]

EZH2 = enhancer of zeste homolog 2; HER2 = human epidermal growth factor receptor 2; HDAC = histone deacetylase; RAIR-DTC = radioactive iodine-refractory differentiated thyroid cancer; *MAPK* = mitogen-activated protein kinase; MEK = mitogen-activated protein kinase.

**Table 5:** Ongoing trials evaluating immunotherapy in RAIR-DTC patients.

Trial	Trial Phase	Patients/Diagnosis	Drug or Drugs Combination	Drug Targets	Status
NCT04061980	II	BRAF V600-positive metastatic and RAIR-DTC	encorafenib + binimetinib ± nivolumab	BRAF, MEK with or without PD-1	Recruiting
NCT02973997	II	lenvatinib-naïve with progressive RAIR-DTC	lenvatinib + pembrolizumab	VEGFR and PD-1	Active, not recruiting
NCT02614495	II	advanced MTC and RAIR-DTC	sulfatinib	VEGFR, FGFR-1, and CSF1R	Recruitment completed
NCT03914300	II	advanced MTC and RAIR-DTC with cancer progression after one VEGFR-treatment	cabozantinib + nivolumab + ipilimumab	VEGFR, PD-1, and CTLA-4	Recruitment suspended
NCT04560127	II	RAIR-DTC	camrelizumab + apatinib	PD-1, VEGFR2/KDR	Recruiting
NCT04544111	II	RAIR-DTC	spartalizumab, + trametinib/dabrafenib	PD-1, MEK-1 and MEK-2, BRAF	Recruiting
NCT03732495	II	bone metastatic RAIR-DTC	lenvatinib + denosumab	VEGFR and RANKL	Recruiting
